# The influence of shunting left/right portal vein branch on post-TIPS hepatic encephalopathy: a study protocol for multicenter randomized blinded controlled trial

**DOI:** 10.1186/s13063-023-07326-9

**Published:** 2023-05-06

**Authors:** Jingqin Ma, Jianjun Luo, Wen Zhang, Yongjie Zhou, Zihan Zhang, Minjie Yang, Zhiquan Zhuang, Li Ma, Jiaze Yu, Xin Zhou, Zhiping Yan

**Affiliations:** 1 Department of Interventional Radiology, Zhongshan Hospital, Fudan University, Shanghai, China; 2 Shanghai Institution of Medical Imaging, Shanghai, China; 3 National Clinical Research Center for Interventional Medicine, Shanghai, China; 4 Department of Center for Tumor Diagnosis and Therapy, Jinshan Hospital of Fudan University, Shanghai, China

**Keywords:** Transjugular intrahepatic portosystemic shunt (TIPS), Hepatic encephalopathy (HE), Randomized controlled trial (RCT)

## Abstract

**Introduction:**

Gastroesophageal varices (GOV) bleeding is a common and serious complication of advanced liver cirrhosis with a median survival of less than 2 years. Multiple guidelines have pointed out that transjugular intrahepatic portosystemic shunt (TIPS) is the rescue treatment of acute variceal hemorrhage (AVB) after failure of standard therapy and an effective second-line treatment for preventing patients with high risks from rebleeding of GOV. The safety and stability of TIPS have been greatly improved due to the improvements of related technologies and the emergence of various novel devices, but the incidence of hepatic encephalopathy (HE) after shunting (10–50%) hindered the widespread use of TIPS. The target portal vein branch might affect the incidence of HE after TIPS. The aim of this study is to compare the rate of HE in patients with hepatitis B virus (HBV) related cirrhosis receiving TIPS either the left or right branch of the portal vein with 8mm Viatorr stent for preventing rebleeding from GOV.

**Methods and analysis:**

This study is a multicenter randomized controlled trial comparing the influence of shunting left or right portal vein branch on post-TIPS hepatic encephalopathy for preventing rebleeding from GOV in patients with HBV-related cirrhosis. A total of 130 patients will be recruited over a period of 24 months across 5 centers in China. Eligible patients will be stratified 1:1 to constructing either a left or right portal vein shunt with an 8-mm Viatorr stent. The primary objective was to compare the incidence of post-TIPS hepatic encephalopathy between the two groups. The secondary objectives were to compare the grade and duration of hepatic encephalopathy, the rate of shunt dysfunction, the rate of variceal rebleeding, the HE-free survival, the cumulative patency rate of the stent, and the overall survival at 12 months and 24 months between two groups.

**Ethics and dissemination:**

This study was approved by the ethics committee of Zhongshan Hospital of Fudan University (No. B2018-292R) and was registered at ClinicalTrials.gov (NCT03825848). All participants give written informed consent.

**Trial registration:**

ClinicalTrials.gov NCT03825848. Registered on January 31, 2019

**Trial status:**

The first patient was recruited into our study on June 19, 2019. A total of 55 patients were recruited till May 27, 2021 (27 and 28 patients assigned to shunting the left (L Group) and right (R Group) branches of the portal vein, respectively).

## Introduction

Gastroesophageal varices (GOV) bleeding is a common and serious complication of advanced liver cirrhosis with a median survival of less than 2 years. Despite the continuous improvement of treatments, the mortality rate within 6 weeks after GOV bleeding is as high as 15–25% [1]. Multiple guidelines have pointed out that transjugular intrahepatic portosystemic shunt (TIPS) is the rescue treatment of acute variceal hemorrhage (AVB) after failure of standard therapy and an effective second-line treatment for preventing patients with high risks from rebleeding of GOV [1–4].

The safety and stability of TIPS have been greatly improved due to the improvements of related technologies and the emergence of various novel devices, but the incidence of hepatic encephalopathy (HE) after shunting (10–50%) hindered the widespread use of TIPS [5]. Post-TIPS HE is defined as a brain dysfunction caused by TIPS, it manifests as a wide spectrum of neurological or psychiatric abnormalities ranging from subclinical alterations to coma [6, 7]. None of the administration of aspartic acid/ornithine [8], lactulose [9], or albumin [10] has been proven to effectively prevent the occurrence of HE after TIPS.

On the other hand, the target portal vein branch might affect the incidence of HE after TIPS. The anatomical study suggested that the blood of the portal vein is composed of superior mesenteric venous blood rich in intestinal toxin and splenic venous blood which is relatively. The laminar flow effect may cause the majority of splenic venous blood flow to enter the left branch of the portal vein while the majority of superior mesenteric vein blood flow enters the right branch of the portal vein. An experimental study also found that the blood ammonia concentration in the right branch of the rabbit portal vein was significantly higher than that in the left branch. Some researchers observed that compared with shunting the right branch of the portal vein, the incidence of HE after shunting the left branch was significantly reduced (15.4~28.7% vs 37.5~56.0%) [11–13]. However, the current TIPS-specific stent is applied in none of the above clinical studies, the diameter of the implanted stent is not uniform, the indications for the patients receiving TIPS treatment are different, and the definition of HE after shunting is inconsistent.

To the authors’ knowledge, no controlled, prospective trials have been published to confirm the influence of shunting the left or right branch portal vein branch on post-TIPS HE. For this reason, the Influence of Shunting left/right portal vein branch on post-Transjugular Intrahepatic Portosystemic shunt Hepatic Encephalopathy (INSTIPHE) has been designed as a prospective, randomized, single-blinded, multicenter trial to compare the rate of HE in patients with hepatitis B virus (HBV) related cirrhosis receiving TIPS either the left or right branch of the portal vein with an 8-mm Viatorr stent for preventing rebleeding from GOV. Secondary objectives include comparisons of the grade and duration of hepatic encephalopathy, the rate of shunt dysfunction, the rate of variceal rebleeding, the HE-free survival, the cumulative patency rate of the stent, and the overall survival at 24 months between two groups.

## Methods

### Study design

**Fig. 1:**
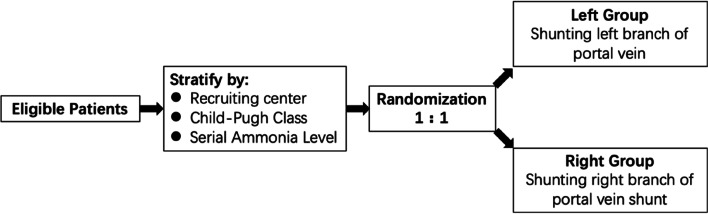
Overview of INSTIPHE trial design

This study is a randomized, controlled, single-blind, multicenter superiority trial with two parallel groups, comparing the influence of shunting the left or right branch of the portal vein on post-TIPS hepatic encephalopathy in patients with HBV-related cirrhosis. A total of 130 patients will be recruited over a period of 24 months across 5 centers in China. Centers will be chosen based on their potential to recruit a high number of patients and their expertise in TIPS treatment and will receive special training with standard TIPS procedures and HE assessment. Eligible patients will be stratified 1:1 to constructing either a left or right portal vein shunt with an 8-mm Viatorr stent (Fig. 1).

This study is conducted in accordance with the Declaration of Helsinki and current good clinical practice guidelines. This study is approved by the ethics committee of Zhongshan Hospital of Fudan University (No. B2018-292R) and was registered at ClinicalTrials.gov (NCT03825848). All other participating centers have obtained the relevant ethics committee approval before patient enrolment.

### Inclusion and exclusion criteria

The inclusion and exclusion criteria for the INSTIPHE trial are summarized in Table 1.

**Table 1:** Patient eligibility criteria for INSTIPHE trial

Inclusion criteria	Exclusion criteria
• Written informed consent	• Imaging confirmed portal vein thrombosis
• Aged 18–75 years old	• History of overt hepatic encephalopathy
• Clinically or histologically diagnosed HBV-related cirrhosis by clinical presentations, laboratory tests, images, and liver biopsies)	• History of TIPS or shunt surgery (including splenectomy, surgical disconnection, or shunt)
• History of GOV bleeding confirmed by endoscopy	• Serial ammonia concentration ≥ 100μmol/L
• Rebleeding of GOV after receiving non-selective β-blockers (NSBBs) combined with endoscopic therapy	• Severe liver insufficiency (total bilirubin > 51μmol/L)
• Child-Pugh A or B	• Consolidation of refractory ascites
• Imaging (CT or MRI) suggests that the left/right first branch portal vein of the intrahepatic portal can construct a shunt	• Malignancy or other serious medical illness that may reduce life expectancy
• Adequate hematological function: Neutrophils ≥1500/mm^3^, Platelets ≥50,000/mm^3^, Prothrombin Time (PT) does not exceed the upper limit of normal control for 3 seconds	• Pulmonary artery pressure > 40 mmHg, left ventricular ejection fraction < 50%, congestive heart failure or other severe cardiopulmonary diseases
• Adequate kidney function: Creatinine <150 μmol/L	• Uncontrolled systemic infection or sepsis

### Recruitment and informed consent

We select potential patients strictly according to the indications for TIPS, and introduce our program to patients in detail through posters and videos in our clinics, to attract them to our program. All relevant information regarding the clinical trial is included in informed consent forms in the Chinese language. Furthermore, the principal investigators will provide a detailed explanation of this trial to the eligible patients. Informed consent must be signed by all patients or their relatives if the informed consent cannot be signed by the patients themselves. All patients’ personal data and medical information will be kept confidential. All patients will be permitted to withdraw from this trial at any time.

### Randomization

Eligible patients will be randomized 1:1 into shunting the left (L Group) or right (R Group) branch of the portal vein. The randomization will be stratified by center, Child-Pugh class (Child-Pugh Class A=5–6 points or Child-Pugh Class B=7–9 points), and the serial ammonia level (lower level ≤ 50 µmol/L or higher level >50 µmol/L) before TIPS. The list will be balanced by different-sized blocks and randomly alternated. The treatment assignment of each eligible patients was generated by the web-based system and was contained in opaque envelopes. This randomization procedure was performed by the coordinator, who was isolated from data collection and analysis. The data coordination center of Zhongshan Hospital will prepare the randomization. Patients and clinicians will not be blinded to the shunting branch of the portal vein in TIPS, so the investigators who perform data evaluation and analysis will not participate in the randomization and treatment.

### Protocol treatment

All enrolled patients received TIPS within 72 h of randomization. After transhepatic puncture of a secondary branch of the intrahepatic portal vein under ultrasound guidance, a 5-F Pigtail catheter will be introduced into the splenic vein and superior mesenteric vein (SMV) to evaluate the index varices as well as the feeding vessels and draining veins. The main feeding vessel (e.g., left, short, or posterior gastric vein) will be embolized with coils and N-butyl-2 cyanoacrylate (NBCA). Portal vein pressure and 5ml blood sample for serial ammonia concentration detection will be obtained at the splenic vein, SMV, main portal vein, and primary branch of the intrahepatic portal vein separately. According to the randomization, the tip of the pigtail catheter will be placed at either the left (L Group) or right (R Group) branch of the portal vein (0.5 cm from the portal vein bifurcation) as a marker for conventional TIPS creation. Viatorr-covered stents (W. L. Gore & Associates, USA) with a diameter of 8 mm and a length of 6–10 cm will be employed in our study. Postoperative portal vein pressure will be measured at the main portal vein level. The transhepatic tract will be embolized with coils or NBCA after TIPS.

Low protein diet, intravenous L-ornithine-L-aspartate (20g qd) with branched-chain amino acids, and oral lactulose (10ml tid) for 3 days will be routinely prescribed for the prevention of post-TIPS hepatic encephalopathy after TIPS.

### Trial assessments

The last enrolled patient will be followed up for 24 months after the start of treatment (D0). All other patients will be followed up until the final visit of the last enrolled patient. Patients will therefore be followed up for a maximum of 48 months and a minimum of 12 months following the start of treatment. All patients will be assessed by the schedule summarized in Table 2.

**Table 2:** Trial assessment schedule

	Enrollment	Treatment			Post-TIPS follow-up					
Evaluation/examination	≤14 days before randomization	D0	D1	D2	M1	M3	M6	M12	M18	M24
Informed consent	×									
Demographics	×									
Medical History	×									
Enhanced abdominal CT	×			×		×		×		×
Ultrasound Elastography	×			×	×	×	×	×	×	×
Upper gastrointestinal endoscopy	×				×	×	×	×	×	×
Hematology^a^	×	×	×		×	×	×	×	×	×
Biochemistry^b^	×		×		×	×	×	×	×	×
Serial ammonia concentration	×		×		×	×	×	×	×	×
Tumor markers^c^	×		×		×	×	×	×	×	×
HBV-DNA	×		×		×	×	×	×	×	×
Child-Pugh	×		×		×	×	×	×	×	×
MELD^d^	×		×		×	×	×	×	×	×
ECOG^e^	×	×	×	×	×	×	×	×	×	×
HE assessment^f^	×	×	×	×	×	×	×	×	×	×
Adverse events	×	×	×	×	×	×	×	×	×	×
Survival	×	×	×	×	×	×	×	×	×	×

^a^Measurement of red blood cells, hemoglobin, white blood cells, absolute neutrophils and absolute lymphocytes,and platelets

^b^Measurement of total bilirubin, direct and indirect bilirubin, albumin, alanine aminotransferase, aspartate aminotransferase, alkaline phosphatase, glutamine transferase, urea nitrogen, serum creatinine, potassium, sodium, prothrombin time, INR, and D-dimer

^c^Measurement of AFP, CEA, and CA-199

^d^Model for End-stage Liver Disease scores

^e^Eastern Cooperative Oncology Group

^f^Measured by Stroop Test and clinical examination

Additional non-study assessments are permitted at the discretion of the treating investigator. Follow-up will be discontinued if the patient receives liver transplantation, if the patient withdraws consent, or if a physician considers it necessary for the patient to discontinue the trial from a medical perspective.

### Outcome measures

The primary endpoint of the INTIPHE trial is to compare the rate of post-TIPS overt hepatic encephalopathy between patients with left or right shunt at 24 months.

The secondary endpoints include comparing the grade and duration of hepatic encephalopathy, covert HE, the rate of shunt dysfunction, the rate of variceal rebleeding, the HE free survival, the cumulative patency rate of stent and the overall survival at 24 months between two groups.

### Outcome definitions

The grade of HE was classified into covert HE (minimal HE and grade I) and overt HE (grade II to grade IV) according to the severity of manifestation (Table 3). Stroop Test (EncephalApp developed by Jasmohan Bajaj) will be used to detect the minimal HE. An off-time and on-time value >190 s will identify a patient with covert HE. Grade I to grade IV HE will be diagnosed by clinical examination. The starting time, duration, treatment, and outcome of every episode will be recorded.

**Table 3:** WHC and clinical description of HE

WHC	ISHEN	Description
Minimal	Covert	Psychometric or neuropsychological alterations of tests exploring psychomotor speed/executive functions or neurophysiological alterations without clinical evidence of mental change
Grade I		Trivial lack of awareness Euphoria or anxiety Shortened attention span Impairment of addition or subtraction Altered sleep rhythm
Grade II	Overt	Lethargy or apathy Disorientation for time Obvious personality change Inappropriate behavior Dyspraxia Asterixis
Grade III		Somnolence to semi stupor Responsive to stimuli Confused Gross disorientation Bizarre behavior
Grade IV		Coma

HE-free survival (HEFS) is defined as the time from D0 to the first episode of HE.

Shunt dysfunction will be suspected in the condition of rebleeding and/or the maximum flow velocity within the shunt is less than 0.5 m/s or absent on color Doppler ultrasound (CDUS). Shunt will be further confirmed by portography if the shunt stenosis is greater than 50% and/or portal-systemic gradient (PSG) is beyond 15 mmHg, and TIPS revision by balloon angioplasty and additional stent-placement will be planned.

Variceal rebleeding is defined as bleeding from gastroesophageal varices confirmed by upper gastrointestinal endoscopy.

### Adverse events and harms

All adverse events occurring after entry into the study will be recorded. The interventions and treatments were performed, if necessary.

### Provisions for post-trial care

If the disease progression will be correlated with our trial, they will receive free treatment and a certain amount of compensation.

### Auditing plan

The clinical research center of Zhongshan Hospital will audit trial conduct every 6 months, which will be independent from investigators.

### Sample size calculation

There is currently no multi-center RCT study on comparing the rate of HE between HBV-related cirrhosis patients with shunting left/right portal vein branches. The sample size was determined on the basis of three single-center study [11–13]: the rate of HE in the patients with left branch shunt was 19.4% (2 years after TIPS), 15.4% (2 years after TIPS) and 22.6% (1 year after TIPS), while the rate of HE in the patients with right branch shunt was and 43.8% (2 years after surgery), 37.5% (2 years after surgery), and 28.6% (1 year after surgery). We presume that the rate of HE after 2 years of TIPS shunting left and right branches will be 16.9% and 33.1%, respectively. Considering a power of 80% with a bilateral alpha risk of 5% and a dropout rate of 10%, we aimed to enroll 130 patients.

### Data management

Clinical coordinators enter raw data from patient records into Case Report Form (CRF) in a timely and complete manner. Consistency of records in CRFs with original information regularly checked by inspectors. If the data analysts have questions about the data, the principal investigator will review the data in CRFs. All patients’ personal data and medical information will be kept confidential, and all participants will be assigned unique identifiers.

### The interim analysis

An interim analysis is performed on the primary endpoint when 50% of patients have been randomized and have completed the 12-month follow-up. The interim analysis is performed by an independent statistician, blinded for the treatment allocation. The statistician will report to the principal investigator, who makes the final decision to terminate the trial.

### Dissemination policy

The results of the study will be released to the public via peer-reviewed publication or academic reports.

### Statistical analysis

All data will be analyzed on the intention-to-treat population and are supplemented by per-protocol set analyses. The intention-to-treat population contains all patients who will undergo randomization (irrespective of whether they successfully receive TIPS), while per-protocol set analyses only include patients who successfully receive TIPS. Continuous variables will be summarized as mean ± standard deviation or the median values (ranges) and will be compared using the independent sample *t* test or one-way analysis of variance. Categorical variables will be expressed as frequencies and compared using the Chi-Square or Fisher’s exact tests. The missing data were performed with multiple imputation by using the MICE procedure. The time-to-event primary and secondary outcomes will be calculated by the Kaplan-Meier method and compared by the log-rank test (Mantel-Haenszel version). Data will be censored when any of the following events occur : liver transplantation, death, end of follow-up time. Estimates of treatment effect (with 95% CIs) were evaluated by using Cox regression models. Two-tailed *p* values <0.05 will be considered statistically significant. All statistical calculations will be performed using SPSS V.25.0 (Chicago, IL, USA).

## Discussion

### Study implications

The high rate of HE, including cover and overt HE, after TIPS hindered the widespread use of TIPS. Although the mechanism of post-TIPS HE is not thoroughly understood, the intestinal toxin from SMV might be the most important cause. However, no randomized controlled studies have evaluated whether the laminar flow of portal vein is affecting the rate of post-TIPS HE. The INTIPHE trial will be the first RCT to compare the rate of post-TIPS HE of shunting the left portal vein branch with that of shunting the right portal vein branch in patients with HBV-related cirrhosis. The severity and duration of HE, the HE-free survival and the overall survival at 12 months and 24 months will also be compared between two groups. If shunting the left portal vein branch does reduce the rate of post-TIPS HE compared to shunting the right portal vein branch, it might be recommended as the standard procedure of TIPS in those patients with a high risk of HE. The results from the INTIPHE trial should further the understanding of the influence of shunting the left/right portal vein branch on post-TIPS HE and determine the optimal treatment strategy of TIPS in patients with HBV-related cirrhosis for preventing rebleeding from GOV.

### Study limitations

Some aspects of the INTIPHE study design are worth further discussion. First, patients with HBV-related cirrhosis, which is the most common cause of liver cirrhosis in China instead of alcohol abuse in Western countries, are our target population. The difference in the etiology of liver cirrhosis might influence the application of our findings in Western countries. Second, eligible patients in the INTIPHE study will be stratified according to the Child-Pugh score as this is an independent prognostic factor of post-TIPS HE. In addition, patients will be stratified by serial ammonia concentration since it is associated with post-TIPS HE despite not being a valid tool to diagnose HE. Third, the rate of post-TIPS HE has been chosen as the primary endpoint as the episode of HE is a better quantitative indicator than the severity or duration of HE. Fourth, an 8-mm Viatorr covered stent was employed in the INTIPHE trial as recent studies found that compared to the 10mm covered stent, implantation of an 8-mm covered stent significantly reduced the incidence of HE after TIPS without affecting the shunt efficiency. Fifth, while blinding to the physicians is not possible due to the treatment methods, the potential biases caused by the lack of physician blinding will be minimized by appointing an independent group of physicians performing the blind follow-up and diagnosis of post-TIPS HE.

## Data Availability

The datasets of the patients used and analyzed in the trial cannot be made publicly available and are available from the corresponding author on reasonable request. Ethics approval and consent to participate This study is conducted in accordance with the Declaration of Helsinki and current good clinical practice guidelines. This study is approved by the ethics committee of Zhongshan Hospital of Fudan University (No. B2018-292R) and was registered at ClinicalTrials.gov (NCT03825848). All other participating centers have obtained the relevant ethics committee approval before patient enrolment. All relevant information regarding the clinical trial is included in informed consent forms in the Chinese language. Further, the principal investigators will provide a detailed explanation of this trial to the eligible patients. Informed consent must be signed by all patients or their relatives if the informed consent cannot be signed by the patients themselves. All patients will be permitted to withdraw from this trial at any time.
